# Biological characteristics and treatment outcomes of metastatic or recurrent neuroendocrine tumors: tumor grade and metastatic site are important for treatment strategy

**DOI:** 10.1186/1471-2407-10-448

**Published:** 2010-08-23

**Authors:** Su-Jung Kim, Jin Won Kim, Sae-Won Han, Do-Youn Oh, Se-Hoon Lee, Dong-Wan Kim, Seock-Ah Im, Tae-You Kim, Dae Seog Heo, Yung-Jue Bang

**Affiliations:** 1 Department of Internal Medicine, Seoul National University Hospital, Seoul National University College of Medicine, Seoul, Republic of Korea; 2 Cancer Research Institute, Seoul National University College of Medicine, Seoul, Republic of Korea

## Abstract

**Background:**

Studies about the biology, treatment pattern, and treatment outcome of metastatic/recurrent neuroendocrine tumor (NET) have been few.

**Methods:**

We enrolled patients with metastatic/recurrent NET diagnosed between January 1996 and July 2007 and retrospectively analyzed.

**Results:**

A total of 103 patients were evaluated. Twenty-six patients (25.2%) had pancreatic NET, 27 (26.2%) had gastrointestinal NET, 2 (1.9%) had lung NET, 28 (27.2%) had NET from other sites, and 20 (19.4%) had NET from unknown origin. The liver was the most common metastatic site (68.9%). Thirty-four patients had grade 1 disease, 1 (1.0%) had grade 2 disease, 15 (14.6%) had grade 3 disease, 9 (8.7%) had large cell disease, and 7 (6.8%) had small cell disease.

Sixty-six patients received systemic treatment (interferon, somatostatin analogues or chemotherapy), 64 patients received local treatment (TACE, radiofrequency ablation, metastasectomy, etc.). Thirty-six patients received both systemic and local treatments.

Median overall survival (OS) was 29.0 months (95% confidence interval, 25.0-33.0) in the103 patients. OS was significantly influenced by grade (*p *= .001). OS was 43.0, 23.0, and 29.0 months in patients who received local treatment only, systemic treatment only, and both treatments, respectively (*p *= .245). The median time-to-progression (TTP) was 6.0 months. Overall response rate was 34.0% and disease-control rate was 64.2%. TTP was influenced by the presence of liver metastasis (*p *= .011).

**Conclusions:**

OS of metastatic/recurrent NET was different according to tumor grade. TTP was different according to metastasis site. Therefore, development of optimal treatment strategy based on the characteristics of NET is warranted.

## Background

In 1890, Ransom described a patient with a carcinoid syndrome and liver metastasis, which was the first report of metastatic neuroendocrine tumor (NET) [1]. Radical surgery has been the only available cure for NETs although more than 50% of these tumors are unresectable at diagnosis. And once metastasis presents, NET is usually not curable with their clinical courses being diverse from relatively indolent to aggressive.

In the case of unresectable metastatic NETs, they has been treated with either local treatment modalities or systemic treatment modalities according to location and burden of metastasis or tumor biology [2]. Systemic treatment including interferon (IFN)-α, somatostatin analogues, and chemotherapy mainly with streptozotocin has been considered palliative and shown only modest antitumor activities [3,4]. Therefore, local treatment modalities such as palliative surgery, transcatheter arterial chemoembolization (TACE), and radiofrequency ablation (RFA) have been frequently utilized in the metastatic setting, especially for liver metastasis.

Because metastatic/recurrent NET is a rare disease, randomized controlled trials have been lacking. However, incidence of NET is increasing according to the recent US Surveillance, Epidemiology, and End Results (SEER) report which is the largest population-based study to date [5]. Recently with an increased understanding of the biology of NETs, it has been possible to actively investigate novel agents and treatments including targeted therapy with some of them proven to be effective [6,7].

Many studies for malignant NETs were done mainly in Western countries while only few have been done in Asian countries. Besides, studies about the biology, treatment pattern, and treatment outcome of metastatic/recurrent NET have been few and far between other than early-stage NET. Therefore we conducted this study to reveal the biologic characteristics and treatment outcomes of metastatic/recurrent NET in a referral center in an Asian country.

## Methods

We consecutively enrolled patients with histologically confirmed metastatic/recurrent NET between January 1996 and July 2007 at Seoul National University Hospital. Medullary carcinoma of thyroid, pheochromocytoma, paraganglioma, small-cell and large-cell neuroendocrine carcinoma of the lung, and adrenal cortical carcinoma were excluded in this analysis because those have unique characteristics. We retrospectively analyzed the characteristics of these population, treatment pattern, and treatment outcomes.

Data about stage before progression to metastatic disease or recurrence was obtained. A localized NET was defined as an invasive neoplasm confined entirely to the organ of origin. A regional NET was defined as a neoplasm that extended beyond the limits of the organ of origin directly into surrounding organs or tissue or a neoplasm involving regional lymph nodes. Finally, a metastatic NET was defined as a neoplasm that spread to parts of the body remote from the primary tumor [5].

There has been no established uniform grading system for NETs. We classified "carcinoid tumors" or "islet cell tumors" or well differentiated tumors into grade 1, atypical carcinoid or moderately differentiated tumors into grade 2, poorly differentiated tumors into grade 3, and anaplastic tumors into grade 4 according to the SEER [5]. In addition, there were large-cell and small-cell neuroendocrine carcinomas from sites other than the lung and from unknown primary origin. These were also included in our analysis.

To evaluate response to systemic treatment, RECIST (response evaluation criteria in solid tumors) criteria were applied. Statistical analyses were performed using the chi-square and the Fisher's exact test to compare response of systemic treatment. The Kaplan-Meier method was used to estimate overall survival (OS) and the time-to-progression (TTP) after systemic treatment. In multivariate analyses for survival, Cox regression analysis was used. Statistical significance was achieved if the probability was less than 5% (*p *< .05). We received approval for this study from the Institutional Review Board of Seoul National University Hospital (IRB No. H-0809-039-256).

## Results

### (1) Baseline characteristics

Table 1 includes the baseline characteristics for the whole population. A total of 103 patients were enrolled. The median duration of follow-up was 40.0 months (range, 0.0-159.0). The median age was 54 years (range, 22-78). There were 58 (56.3%) males.

**Table 1:** Baseline characteristics of 103 patients

Characteristic			Frequency	%
	Median (years)		54 (range, 22-78)	
Age	<60		63	61.2
	≥ 60		40	38.8

Sex	Male		58	56.3
	Female		45	43.7

	Absent		92	89.3
Carcinoid symptom	Present		5	4.9
	Unavailable		6	5.8

	Pancreas		26	25.2
	GI tract		27	26.2
	Foregut		11	10.7
	Midgut		1	1.0
Origin	Hindgut		15	14.6
	Lung		2	1.9
	Etc.*		28	27.2
	Unknown		20	19.4

Initial metastatic site	Liver		50	74.6
	Bone		12	17.9
	Lung		12	17.9
	Brain		3	4.5
	Lymph node		20	29.9
	Etc.		10	14.9

Recurrent site	Liver		15	41.7
	Bone		16	44.4
	Lung		1	2.8
	Brain		1	2.8
	Lymph node		15	41.7
	Etc.		3	8.3

	Local		21	21.4
Prior stage	Regional		15	14.6
	Distant		67	65.0

	1		34	33.0
	2		1	1.0
Grade	3		15	14.6
	Large		9	8.7
	Small		7	6.8
	Unclassified		25	24.3
	Unavailable		12	11.7

		Median (range)	23.0 (2.6-1324.6)	
	24-hour urine 5-HIAA (μmol/day)^†^	<31.4 (reference range)	15	71.4
Biomarkers		≥ 31.4	6	28.6
		Median (range)	1.6 (0.4-23.1)	
	Serum NSE (nmol/l)^‡^	<1.0 (reference range)	12	36.4
		≥ 1.0	21	63.6

GI, gastrointestinal; 5-HIAA, 5-hydroxyindoleacetic acid; NSE, neuron-specific enolase

* The biliary tract (5 cases), thymus (4), uterus (4), mediastinum (3), oral cavity (2), skin (2), trachea (1), liver (1), kidney (1), pelvis (1), prostate (1), bladder (1), nasal cavity (1), and orbit (1)

^† ^Available data: 21

^‡ ^Available data: 33

Twenty-six patients (25.2%) were diagnosed with pancreatic NET, 27 patients (26.2%) with GI NET, and 2 patients (1.9%) with lung NET. Tumors that originated from sites other than the pancreas, GI tract, and lung were observed in 28 patients (27.2%). There were 20 patients (19.4%) of whom the origin of the tumor was unknown. In GI NET, 11 cases (40.7%) were foregut NET, 1 case (3.7%) was midgut NET, and 15 cases (55.6%) were hindgut NET. As an initial site of metastasis, the liver was the most common site (50 patients, 74.6%). As a site of recurrence, the liver was also the most common site (16, 44.4%).

Sixty-seven patients were diagnosed with metastatic disease from their initial diagnosis, 30 patients had recurrent disease after curative resection and 6 patients had disease which progressed to metastatic disease from a non-metastatic disease state after initial diagnosis.

Regarding the grade classification of NET, 34 patients (33.0%) had grade 1 disease, 1 patient (1.0%) had grade 2 disease, 15 patients (14.6%) had grade 3 disease, 9 patients had (8.7%) large cell disease, and 7 patients (6.8%) had small cell disease. Grade was unclassified in 25 patients (24.3%) and information about grade was unavailable in 12 patients (11.7%).

At the time of diagnosis of metastatic/recurrent disease, the median value of 5-hydroxyindoleacetic acid (5-HIAA) from 24-hour urine samples was 23.0 μmol/day (range, 2.6-1324.6) and the median value of serum levels for neuron-specific enolase (NSE) was 1.6 nmol/l (range, 0.4-23.1).

### (2) Treatment outcomes and prognostic factors of metastatic/recurrent NET

**Figure 1:**
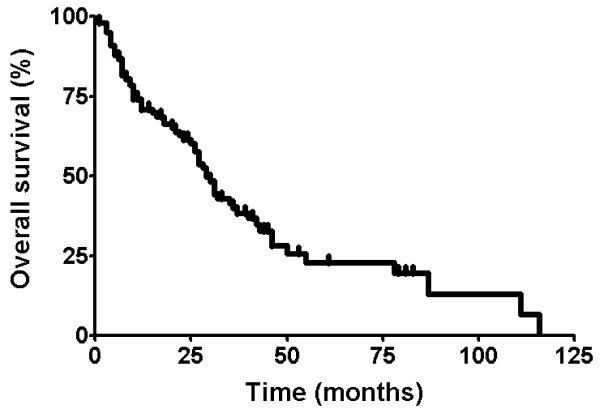
Overall survival in 103 patients

**Figure 2:**
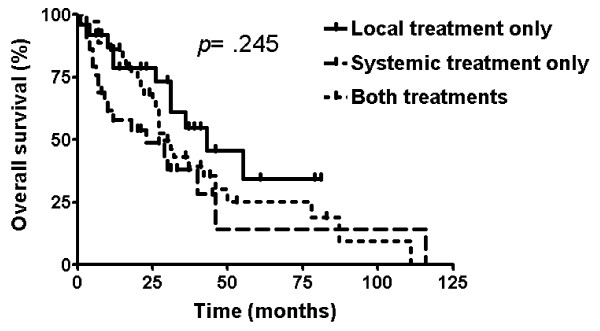
Overall survival by treatment modality

Figure 1 shows OS in all of the 103 patients. Median OS was 29.0 months (95% confidence interval [CI], 25.0-33.0). The three-year survival rate was 39.2%. Table 2 shows OS according to characteristics. Survival was not significantly different by age (*p *= .053), sex (*p *= .461), carcinoid symptom (*p *= .646), primary tumor origin (*p *= .660), presence of liver metastasis (*p *= .995), elevation of biomarkers (*p *= .653 for urine 5-HIAA; *p *= .051 for serum NSE), and treatment modality (local treatment only vs. systemic treatment only vs. both treatments, *p *= .245). Figure 2 illustrates OS according to treatment modality.

**Table 2:** Overall survival according to characteristics (n = 103)

Characteristic			Median OS	95% CI	3-year survival rate (%)	*P*-value
Age	<60 ≥ 60		32.0 27.0	20.9-43.1 12.6-41.4	44.2 31.0	.053

Sex	Male Female		29.0 31.0	20.5-37.5 26.7-35.3	36.4 42.9	.461

Carcinoid symptom	Absent Present		29.0 28.0	23.3-34.7 23.7-32.3	40.5 -	.646

	Pancreas		43.0	23.4-62.6	45.3	
Origin	GI		40.0	10.3-69.7	51.2	.660
	Lung		10.0	-	-	

Liver metastasis	Absent Present		29.0 29.0	18.6-39.4 22.8-35.2	42.2 38.1	.995

	1		78.0	32.3-123.7	65.8	
Grade	2-3		18.0	1.9-34.1	29.8	.001
	Large		15.0	2.8-27.2	13.9	
	Small		18.0	7.1-28.9	17.9	

	24-hour urine 5-HIAA (μmol/day)	<31.4	31.0	24.3-37.7	42.8	.653
		≥ 31.4	46.0	-	66,7	
Biomarkers						
	Serum NSE (nmol/l)	<1.0	31.0	-	47.7	.051
		≥ 1.0	7.0	4.3-9.7	26.8	

	Local treatment only		43.0	23.1-62.9	53.0	
Treatment modality	Systemic treatment only		23.0	0.4-45.6	38.0	.245
	Both treatments		29.0	23.6-34.4	43.0	

OS, overall survival; CI, confidence interval; GI, gastrointestinal; 5-HIAA, 5-hydroxyindoleacetic acid; NSE, neuron-specific enolase

**Figure 3:**
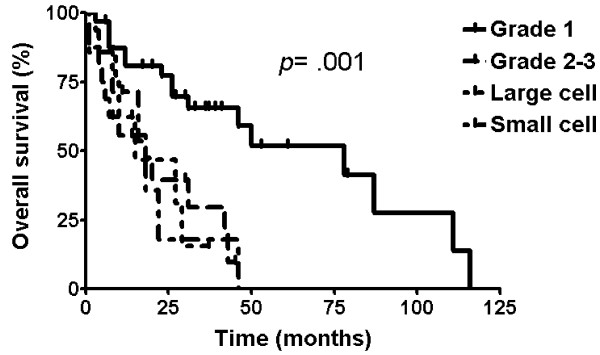
Overall survival by grade

OS was significantly influenced by grade (*p *= .001) (Figure 3). The significance was derived from the difference between grade 1 NET and the others (grade 2/3, large-cell, and small-cell disease) (78.0 months vs. 18.0 months, respectively).

### (3) Patterns of treatment

As an initial treatment of metastatic/recurrent disease setting, systemic treatment was the most common (43 patients, 41.7%), followed by TACE (21, 20.4%), surgery (13, 12.6%), best supportive care (10, 9.7%), endoscopic removal (6, 5.8%), and radiotherapy (4, 3.9%).

In the entire course of the disease, 66 patients received systemic treatment (IFN, somatostatin analogues, and chemotherapy), 64 patients received local treatment to the metastatic/recurrent site (TACE, RFA, metastasectomy, endoscopic removal, and radiotherapy). Thirty-six patients received both of systemic and local treatment and 9 patients received best supportive care only.

### A. Systemic treatment in metastatic/recurrent NET

Among the 103 patients, 66 patients received palliative systemic treatment. Median time from diagnosis of metastatic/recurrent NET to initiation of systemic treatment was 0.0 months (range, 0.0-77.0). The median line of systemic treatment which was administered was the 2 lines (range, 1st-7th). Among 66 patients receiving the 1st-line systemic treatment, 36 patients received 2nd-line systemic treatment, and among them, 22 patients received 3rd-line systemic treatment. Baseline characteristics among the 66 patients who received palliative systemic treatment are demonstrated in Table 3.

**Table 3:** Baseline characteristics among the 66 patients who received palliative systemic treatment

Characteristic			Frequency	%
	Median (years)		54 (range, 22-78)	
Age	<60		42	63.6
	≥ 60		24	36.4

Sex	Male		41	62.1
	Female		25	37.9

	Absent		61	92.4
Carcinoid symptom	Present		3	4.5
	Unavailable		2	3.0

	Pancreas		2	3.0
	GI		15	22.7
Origin	Lung		15	22.7
	Etc.*		22	33.3
	Unknown		12	18.2

	Local		10	15.2
Prior stage	Regional		9	13.6
	Distant		47	71.2

Grade	1		20	30.3
	2		1	1.5
	3		10	15.2
	Large		8	12.1
	Small		5	7.6
	Unclassified		15	22.7
	Unavailable		7	10.6

	24-hour urine 5-HIAA (μmol/day)^†^	Median (range)	33.5 (6.8-341.2)	
		<31.4	5	45.5%
Biomarkers		≥ 31.4	6	54.5%
		Median (range)	1.36 (0.2-23.1)	
	Serum NSE (nmol/l)^‡^	<1.0	8	36.4%
		≥ 1.0	14	63.6%

Abbreviations as in table 1, 2

*The uterus (4 cases), biliary tract (3), thymus (3), mediastinum (2), oral cavity (2), skin (2), trachea (1), kidney (1), prostate (1), bladder (1), nasal cavity (1), and orbit (1)

^† ^Available data: 11

^‡ ^Available data: 22

Median OS for the 66 patients was 25.0 months (95% CI, 18.6-31.4). The three-year survival rate was 32.6%. As a 1st-line systemic treatment, IFN was administered to 15 patients (22.7%), somatostatin-analogue to 2 patients (3.0%), and chemotherapeutic agent to 49 patients (74.2%). Drugs which were administered are shown in Table 4.

**Table 4:** Drugs which were used in the 1st line

Drugs	Number
Interferon	15
Somatostatin-analogues	2
EP, EC	16
CAV	3
TC, TP, DP, DC	4
VIP	6
Adriamycin+streptozotocin	2
FOLFOX, XELOX	2
FP, FC	4
IP	1
5-FU+adriamycin+streptozocin	2
Dacarbazine	1
Sunitinib	5
RAD001	3
Total	66

EP, etoposide and cisplatin; EC, etoposide carboplatin; CAV, cyclophosphamide, adriamycin and vincristine; TC, paclitaxel and carboplatin; TP, paclitaxel and cisplatin; DP, docetaxel and cisplatin; DC, docetaxel and carboplatin; VIP, etoposide, ifosfamide and cisplatin; FOLFOX, 5-fluorouracil (5-FU), leucovorin and oxaliplatin; XELOX, capecitabine, oxaliplatin; FP, 5-FU and cisplatin; FC, 5-FU and carboplatin; IP, irinotecan and cisplatin; RAD001, everolimus

**Figure 4:**
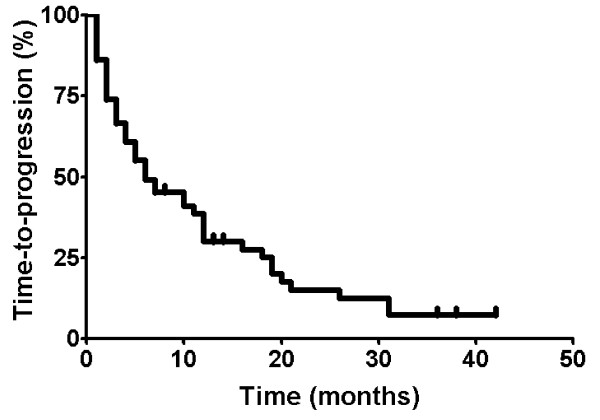
Time-to-progression in 66 patients in the 1st line

The median TTP after 1st-line systemic treatment was 6.0 months (95% CI, 3.3-8.7). One-year progression-free rate was 30.2% (Figure 4). Overall response rate (ORR) was 34.0% (CR, 3.8%; PR, 30.2%; 95% CI, 20.8-47.1) and disease-control rate (DCR) was 64.2% (95% CI, 50.8-77.5) in the 1st-line systemic treatment.

As a 2nd-line systemic treatment, 2 patients (5.6%) received somatostatin-analogues and 34 patients (94.4%) received chemotherapy. The median TTP after 2nd-line systemic treatment was 5.0 months (95% CI, 3.5-6.5). One-year progression-free rate was 14.1%. ORR was 21.4% and DCR was 53.6%.

As a 3rd-line systemic treatment, 1 patient (4.5%) received IFN and 21 patients (95.5%) received chemotherapeutic agents. The median TTP was 2.0 months (95% CI, 1.5-2.5). One-year progression-free rate was 5.5%. ORR was 12.5% and DCR was 31.3%.

Tables 5 and 6 show OS, TTP, ORR, and DCR according to the characteristics of the 66 patients. Although OS was not significantly different according to treatment modality (*p *= .350), OS in patients who received only systemic treatment and not local treatment (23.0 months) seemed to be shorter than the patients who received both local and systemic treatment (29.0 months). OS was different between grade 1 (36.0 months) and the others (17.0 months), although insignificant (*p *= .215).

**Table 5:** Overall survival and time-to-progression (n = 66)

Characteristic			OS* (months)	95% CI	1-year survival rate (%)	*P*-value	TTP (months)	95% CI	1-year survival rate (%)	*P*-value
Age	<60 ≥ 60		26.0 25.0	18.9-33.1 14.2-35.8	69.2 69.9	.656	6.0 6.0	2.8-9.2 0.8-11.2	26.9 35.4	.611

Sex	Male Female		22.0 27.0	15.9-28.1 19.3-34.7	69.1 69.6	.134	6.0 6.0	0.0-14.2 2.7-9.3	28.0 34.6	.771

	Pancreas		28.0	18.0-38.0	77.1	.981	7.0	0.0-15.0	41.6	
Origin	GI		22.0	0.0-46.9	63.2		2.0	0.8-3.2	25.0	.258
	Lung		9.0	-	50.0		1.0	-	-	

Liver metastasis	Absent Present		25.0 22.0	20.8-29.2 13.3-30.7	77.0 65.0	.310	12.0 4.0	9.2-14.8 1.3-6.7	40.7 23.8	.011

	1		36.0	13.2-58.8	72.4		12.0	8.3-15.7	57.5	
	2-3		17.0	1.1-32.9	53.0	.612	4.0	1.2-6.8	34.3	
Grade										.681
	Large		12.0	0.0-29.8	50.0		2.0	0.0-4.5	45.0	
	Small		17.0	10.1-23.9	100.0		12.0	2.6-21.4	30.0	

	24-hour urine 5-HIAA (μmol/day)	<31.4	20.0	0.0-52.2	60.0	.114	1.0	-	-	.045
		≥ 31.4	40.0	-	62.5		12.0	0.0-29.6	44.4	
Biomarkers										
	Serum NSE (nmol/l)	<1.0 ≥ 1.0	25.0 6.0	18.0-32.0 0.0-18.0	75.0 46.4	.170	2.0 4.0	0.9-3.1 1.2-6.8	0.0 22.2	.381

	IFN		36.0	22.6-49.4	86.7	.169	10.0	0.0-25.6	22.9	
Regimen	Somatostatin-analogue^†^		3.0	.	50.0		0.0	-	-	.057
	Chemotherapy		20.0	13.3-26.7	64.5		6.0	3.6-8.4	34.2	

Treatment modality	Systemic treatment only Systemic+local treatment		23.0 29.0	0.4-45.6 23.6-34.4	57.9 86.1	.350				

OS, overall survival; TTP, time-to-progression; CI, confidence interval; 5-HIAA, 5-hydroxyindoleacetic acid; NSE, neuron-specific enolase; IFN, interferon

*OS was estimated after initiation of systemic treatment except in treatment modality where OS was calculated after diagnosis of metastasis.

^†^Somatostatin-analogue was given to only 2 patients.

**Table 6:** Overall response rate and disease-control rate (n = 66)

Characteristic			ORR (%)	*P*-value	DCR (%)	*P*-value
Age	<60 ≥ 60		35.3 31.6	.784	61.8 68.4	.628

Sex	Male Female		30.3 40.0	.470	69.7 55.0	.279

	Pancreas		35.7		71.4	
Origin	GI		30.8	1.000	38.5	.055
	Lung		0.0		0.0	

Liver metastasis	Absent Present		58.8 22.2	.009	82.4 55.6	.072
					

	1		33.3		66.7	
	2-3		44.4		66.7	
Grade				.460		.433
	Large		40.0		40.0	
	Small		33.3		100.0	

	24-hour urine 5-HIAA (μmol/day)	<31.4 ≥ 31.4	0.0 33.3	.455	20.0 66.7	.242
Biomarkers						
	Serum NSE (nmol/l)	<1.0 ≥ 1.0	0.0 55.6	.034	28.6 77.8	.126

	IFN		23.1		53.8	
Regimen	Somatostatin-analogue*		0.0	.400	0.0	.079
	Chemotherapy		39.5		71.1	

ORR, overall response rate; DCR, disease-control rate; GI, gastrointestinal; 5-HIAA, 5-hydroxyindoleacetic acid; NSE, neuron-specific enolase; IFN, interferon

*Somatostatin-analogue was given to only 2 patients.

**Figure 5:**
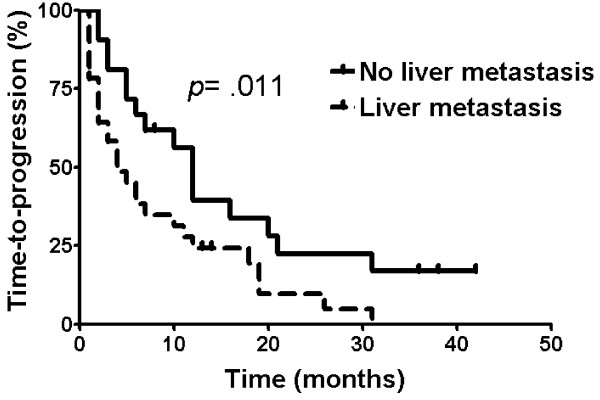
Time-to-progression according to presence of liver metastasis

TTP was influenced by the presence of liver metastasis (*p *= .011). The median TTP in the group without liver metastasis was 12.0 months and in the group with liver metastasis was 4.0 months (Figure 5).

There was a relationship between ORR and the presence of liver metastasis (*p *= .009) or the elevation of serum NSE (*p *= .034). DCR was not significantly related to any clinical factors.

### B. Local treatment in metastatic/recurrent NET

Twenty-five patients received TACE. Median number of TACE was 4 times (range 1-16). Median time interval of TACE was 3.0 months (range, 0.0-72.0). Six patients received RFA. Median number of RFA was 1 time (range 1-2). Metastasectomy was done to 6 patients. Radiotherapy was given to 15 patients except for palliative radiotherapy of bone metastasis. Five patients had their tumors endoscopically removed.

## Discussion

This study presented the characteristics and treatment outcomes of metastatic/recurrent NET. In our study, the origins of metastatic/recurrent NET was found in diverse places in the body and grade 1 disease was the most common (33.0%). Median OS was 29.0 months in all of the 103 cases. Median OS for 66 patients who received palliative systemic treatment was 25.0 months. These were comparable to outcome of the SEER analysis which reported an OS of 25.0 months for metastatic NET among Asians and Pacific islanders. The median TTP after 1st-line systemic treatment was 6.0 months with various regimens. Overall response rate (ORR) was 34.0% and disease-control rate (DCR) was 64.2% in the 1st-line systemic treatment.

The grade has been known to be prognostic in several studies [5,8,9]. OS was significantly influenced by the grade in our study (*p *= .001). Grade 1 tumors showed longer OS than the others. On the contrary, higher grade predicted better response after systemic chemotherapy in several studies [2,10], while not in others [11].

There are a few reports that revealed the similarity of the natural course and treatment outcome between small cell and poorly differentiated neuroendocrine carcinoma [12], or between high-grade non-small cell neuroendocrine carcinoma of lung and small-cell lung cancer [13]. In our study also, grade-3, large cell, and small cell NET did not show any difference in OS.

There has been no established uniform grading system for NETs. Recently WHO (World Health Organization) or ENET (European Neuroendocrine Tumor Society) grading systems are available. WHO suggested a grading system for gastoenteropancreatic NETs. However, the grading system is not compatible with grading system in lung NETs (typical carcinoid, atypical carcinoid, large cell neuroendocrine carcinoma, and small cell lung cancer). ENETS proposed grading systems for foregut, midgut and hindgut NETs those are composed of mitotic count and Ki-67 index. Because our data dealt with NETs from all sites of body and we did not have sufficient number of Ki-67 data, we followed the analysis method used in the SEER report, which included NETs from most sites and classified NETs into 4 grade groups (Grade 1-4). We used it also to compare our results with SEER report.

Presence of liver metastasis was related to shorter TTP (*p *= .011) and lower ORR (*p *= .009) in our study. Presence of liver metastasis tended to be related to a worse response to chemotherapy. A relationship between liver metastasis of NET and response to chemotherapy has rarely been reported before. Further study is required to fully understand the implications of this result. In this case, it suggests a possible role for local treatment modalities in the treatment of NET patients with liver metastasis.

On the contrary, the presence of liver metastasis was not related to OS in our study. The presence of liver metastasis was reported as a negative prognostic factor among all stages of NET in some studies [14], while it has seldom been studied as a prognostic factor among metastatic NETs as in our study.

Urinary 5-HIAA [15] and serum NSE [9] have been well known as a prognostic factor. Patients whose serum NSE level was elevated showed a shorter OS in our study although statistically insignificant (*p *= .051). And elevation of serum NSE (*p *= .034) was related to a higher ORR. More research should be done to further explore this relationship between serum NSE and response to chemotherapy.

In metastatic disease, pancreatic NET has been generally known to have a poor prognosis compared to GI NET [16]. Pancreatic NET has been known to be more chemosensitive than GI NET [3,17]. Comparison of survival and treatment outcome between pancreatic, GI and lung NET in a metastatic setting has rarely been done yet. Treatment outcome after systemic treatment was not statistically different according to the origins of the primary tumor found in the pancreas, GI tract, or lungs in this study.

Patients received either local treatment modalities or systemic treatment modalities or both. Local treatment modalities such as TACE, metastasectomy, endoscopic removal, and radiotherapy were given actively in the course of treatment. Whatever treatment modalities patients received did not influence patients' survival in our study. However, tendency to survive the longest was observed in patients who received local treatment only, followed by patients who received both treatments. In a subgroup analysis within the 3 groups, the systemic treatment group was related to high grade (*p *= .037) and extrahepatic metastasis (*p *= .015) (Table 7).

**Table 7:** Comparison according to treatment modality (n = 63)

		Local treatment	Systemic treatment	Both treatments	*P*-value
Grade	1 2-3 Large Small	14 (73.7%) 3 (15.8%) 1 (5.3%) 1 (5.3%)	7 (38.9%) 8 (44.4%) 3 (16.7%) 0 (O.0%)	13 (50.0%) 3 (11.5%) 5 (19.2%) 5 (19.2%)	.037

Metastatic site	Extrahepatic metastasis Liver metastasis only	7 (33.3%) 14 (66.7%)	17 (77.3%) 5 (22.7%)	11 (52.4%) 10 (47.6%)	.015

Recently in a large retrospective study, the role of surgery was demonstrated in distant pancreatic NET [18]. However, there have been few studies which compared local with systemic treatment modalities or systemic with both treatment modalities in a randomized controlled setting [19].

Regimens used in systemic treatment were diverse in this study. Eleven patients received biotherapy such as IFN and somatostatin analogues as the 1st-line. However, which kind of biotherapy or chemotherapy they received did not have any relationship to treatment outcome and survival in our study.

IFN-α has been used for treatment of patients with NETs for more than 20 years [20,21]. However, its antitumor efficacy has not been satisfactory [22,23]. Somatostatin analogues have been considered mainly an antisecretory drug for symptom control in NET and its ability to control the growth of NET has been a matter of debate [22]. Recently, a result from a randomized controlled trial was reported which demonstrated favorable response and prolongation of TTP after use of somatostatin analogues in well-differentiated midgut NET [24].

The standard chemotherapy for pancreatic NET has been a combination of adriamycin and streptozotocin and to a lesser extent a combination of 5-fluorouracil (5-FU) and streptozotocin [3]. Although 5-FU and streptozotocin have shown modest antitumor effect, there has been no clear standard chemotherapy for carcinoid tumors [23,25]. Some reports have suggested a higher chemosensitivity of undifferentiated or poorly differentiated NET with etoposide-cisplatin combination [2,26].

Recently, several new agents including target agents are actively being tried for advanced NET. Sunitinib showed 16.7% of ORR and 68% of DCR in pancreatic NETs in a nonrandomized study [27]. In a phase II trial, everolimus (RAD001) and octreotide long-acting repeatable (LAR) showed 22% of ORR and 70% of DCR in advanced low to intermediate-grade NETs [7]. Besides these drugs, cytotoxic chemotherapy including a capecitabine-oxaliplatin combination [11] also showed modest antitumor activities in advanced NETs. Radiolabeled somatostatin analogues have been tried actively and showed modest antitumor activities [28,29].

In our analysis, there are several limitations. This was a retrospective study so information such as carcinoid syndrome and biochemical features was not available in all of the patients. And the data pool was heterogeneous and the used regimens were diverse, even though the relative rarity of NETs makes it difficult to collect sufficient numbers of homogenous groups.

Other limitation is that we did not analyze the prognosis and response to systemic treatment according to the Ki67 status of tumors. The Ki67 is being regarded as an important prognostic factor which demonstrates the proliferative capacity of tumors [30]. In our patient pool, there was no available full data on Ki67. Furthermore, we did not have data about serum chromogranin A, of which the clinical meaning and importance are being highlighted nowadays, because this study was a retrospective research composed of patients from 1996. Further study on NET should harbor the contents of Ki67 and chromogranin A.

Nevertheless, this study has several strong points. There have been few reports which dealt with metastatic/recurrent NET as a whole group and showed the treatment outcomes. And we tried to search for predictive factors after palliative systemic treatment. Furthermore, we described the treatment patterns and outcomes in terms of continuum of care. And, as far as we know, this is one of the largest studies which have been done to date with this disease group in Asian countries.

## Conclusions

OS of metastatic/recurrent NET was different according to tumor grade and TTP was different according to metastasis site. Therefore, development of optimal treatment strategy based on the characteristics of NET as well as new active agents is warranted.

## Competing interests

The authors declare that they have no competing interests.

## Authors' contributions

SJK and JWK were involved in collecting and analyzing data and drafting the manuscript. DYO and YJB conceived of the study and participated in its design and coordination. SHJ, DWK, SAI, TYK, and DSH helped to collect the patient pools. DYO and all the other authors read and approved the final manuscript.

## Pre-publication history

The pre-publication history for this paper can be accessed here:

http://www.biomedcentral.com/1471-2407/10/448/prepub
